# The determinants of bootstrap financing in crises: evidence from entrepreneurial ventures in the COVID-19 pandemic

**DOI:** 10.1007/s11187-020-00445-6

**Published:** 2021-01-25

**Authors:** Joern H. Block, Christian Fisch, Mirko Hirschmann

**Affiliations:** 1 Faculty of Management, Trier University, Universitätsring 15, 54296 Trier, Germany; 2 Erasmus School of Economics, Erasmus University Rotterdam, P.O. Box 1738, 3000 Rotterdam, DR Netherlands; 3 Universität Witten/Herdecke, Wittener Institut für Familienunternehmen, Alfred-Herrhausen-Straße 50, 58455 Witten, Germany

**Keywords:** Entrepreneurship, Bootstrap finance, COVID-19, Crises, Coronavirus, G30, L26, M13

## Abstract

Bootstrap financing refers to measures that entrepreneurial ventures undertake to preserve liquidity (e.g., reducing expenses, collecting receivables, delaying payments, preselling). Prior research shows that bootstrap financing is an important enabler for the growth of resource-constrained early-stage ventures. However, little is known about the use of bootstrap financing in crises, during which the preservation of liquidity is particularly salient. We investigate the determinants of bootstrap financing in the 2020 COVID-19 crisis using a sample of 17,046 German entrepreneurial ventures. We formulate hypotheses about the determinants of bootstrap financing from a necessity, human capital, and opportunity cost perspective. Among others, our results show that the severity of the crisis for the venture, the level of private consumption, and self-employment experience are positively associated with an increased use of bootstrap financing measures. Our study contributes to the literature on bootstrap financing and illuminates how entrepreneurial ventures maintain liquidity in crises.

**Plain English Summary** Economic downturns or crises often lead to financial distress for ventures. To survive such tumultuous times, ventures need to preserve their liquidity. Bootstrap financing refers to measures that entrepreneurial ventures take to preserve liquidity (like sending payment reminders, paying invoices later, reducing tax advances, reducing commercial rent). Because little is known about how bootstrap financing is used during crises, we investigate how it was used during the COVID-19 crisis. Our study builds on a survey of 17,046 German entrepreneurial ventures and self-employed individuals. We find that the use of bootstrap financing is positively related to how severe the crisis was for the venture along with the level of private consumption and self-employment experience of the venture’s owner. In contrast, a negative association exists with private liquidity, business liquidity, how long before the owner retires, and part-time self-employment. The positive association between self-employment experience and bootstrap financing indicates that targeted entrepreneurship education programs or webinars should focus on inexperienced entrepreneurs so that these individuals are prepared to use bootstrapping methods to maintain liquidity during crises.

## Introduction

Entrepreneurial ventures engage in bootstrap financing to preserve a critical level of liquidity. Maintaining liquidity is especially important in economic downturns or crises, which often lead to financial distress for ventures (e.g., Ebben 2009; Neeley and Van Auken 2010). Typical measures of bootstrap financing include reducing expenses, collecting receivables more quickly, delaying payments, and preselling (e.g., Winborg and Landström 2001).

While the use of bootstrap financing is an established strategy for entrepreneurial ventures, little is known about the use of bootstrap financing in crises, in which the preservation of liquidity is especially critical for venture survival (e.g., Grichnik et al. 2014). So far, prior research has mainly focused on bootstrapping as a strategic practice for venture growth (e.g., Harrison et al. 2004; Vanacker et al. 2011). Against this background, we address the following research question: Which factors determine the use of bootstrap financing measures during economic crises?

We enrich prior research by investigating the determinants of bootstrap financing during the COVID-19 crises. The 2020 COVID-19 pandemic led to a severe economic downturn that affected most entrepreneurial ventures and their financial situation due to interrupted supply chains, reduced demand, and a generally uncertain environment (Nicola et al. 2020). To address our research question, we use a survey of 17,046 German entrepreneurial ventures and self-employed individuals that was conducted in the middle of the COVID-19 crisis. Conceptually, we formulate hypotheses about the determinants of bootstrap financing from a necessity, human capital, and opportunity cost perspective.

We identify a positive relationship between the severity of the crisis and the use of bootstrapping. In line with our hypotheses, we show that the severity of the crisis for the venture, the level of private consumption, and self-employment experience are positively associated with an increased use of bootstrap financing measures. In contrast, a negative association exists with private liquidity, business liquidity, a shorter time to retirement, and part-time self-employment.

We make three contributions to the literature. First, we contribute to the literature on bootstrap financing in entrepreneurship (e.g., Grichnik et al. 2014; Harrison et al. 2004; Jonsson and Lindbergh 2013; Vanacker et al. 2011; Winborg and Landström 2001). Specifically, we investigate the determinants of bootstrap financing in crises, which is closely related to prior research on the use of internal bootstrapping methods for self-financing (e.g., Grichnik et al. 2014; Winborg and Landström 2001). We contribute to this research by explaining how necessity, human capital, and opportunity cost characteristics influence the use of such internal bootstrap financing methods. Second, we add to previous literature on entrepreneurship in crises (e.g., Brown et al. 2020; Davidsson and Gordon 2016; Fairlie and Krashinsky 2012; Kuckertz et al. 2020; Marino et al. 2008; Snyder 2004; Thorgren and Williams 2020). We show, for example, that the more severe the situation is for the self-employed and their entrepreneurial ventures, the greater the number of bootstrap financing measures used to mitigate the negative consequences of the crisis. Third, we contribute to research linking human capital theory and entrepreneurial finance (e.g., Davidsson and Honig 2003; Liu et al. 2018). While our results on the use of bootstrap financing in crises similarize prior research on entrepreneurial experience and access to capital (e.g., Robinson and Sexton 1994), the results contrast Grichnik et al. (2014) since we do not find support for a positive effect of education on the extent to which bootstrap financing is applied in crises.

## Related literature

### Prior research on bootstrap financing

Prior research has extensively discussed how financial bootstrapping can enable entrepreneurs and small firms to overcome financial constraints (e.g., Carter and Van Auken 2005; Ebben and Johnson 2006; Grichnik et al. 2014; Harrison et al. 2004; Jonsson and Lindbergh 2013; Vanacker et al. 2011; Winborg and Landström 2001). Specifically, financial bootstrapping is used in situations where entrepreneurs or entrepreneurial ventures require (financial) resources without relying on long-term external funding (e.g., Ebben and Johnson 2006; Grichnik et al. 2014; Winborg and Landström 2001). Instead, they acquire resources and preserve liquidity through financial bootstrapping (e.g., Freear et al. 1995; Harrison et al. 2004; Van Auken and Neeley 1996).

Different measures of financial bootstrapping exist. Grichnik et al. (2014) identify customer-related, joint resource utilization, internal self-financing, and temporary resource methods of bootstrapping. Since bootstrap financing in crises is shaped by liquidity bottlenecks, internal self-financing methods of bootstrapping are of major importance. Examples of such internal bootstrapping methods are the following: (1) obtaining loans from relatives, (2) using of own credit card, (3) withholding the entrepreneur’s own salary, (4) delaying payments to suppliers, and (5) delaying payments of taxes (e.g., Grichnik et al. 2014).

Prior research documents differences in financial bootstrapping between smaller and larger firms. Harrison et al. (2004) show that smaller firms use financial bootstrapping to reduce costs, whereas larger firms tend to exploit value-chain-based relationships with customers or suppliers. These findings are consistent with the findings of Ebben and Johnson (2006), who indicate that ventures increase their use of customer-related bootstrapping measures over time. Research also shows that many businesses use financial bootstrapping to decrease the need for external capital, in particular in early venture stages (e.g., Harrison et al. 2004; Kim et al. 2006).

Additionally, prior studies outline different outcomes of use of financial bootstrapping measures (e.g., Grichnik et al. 2014; Jonsson and Lindbergh 2013; Vanacker et al. 2011). First, entrepreneurs or small businesses that rely on financial bootstrapping activities exhibit higher growth over time (e.g., Brush et al. 2006; Vanacker et al. 2011). For example, Vanacker et al. (2011) find that certain bootstrapping measures (e.g., hiring more interim personnel, encouraging customers to pay sooner) lead to growth in the long term. Second, bootstrapping activities help entrepreneurial firms develop social capital (Jonsson and Lindbergh 2013). Also, Grichnik et al. (2014) find that human and social capital lead to higher usage of financial bootstrapping. Therefore, the authors suggest that business training and an extension of the entrepreneurial network can support small businesses and enable them to better overcome financial constraints.

### Bootstrap financing and economic crises

While different measures and outcomes of financial bootstrapping have been widely investigated, little research exists regarding bootstrap financing in crises (e.g., Ebben 2009; Neeley and Van Auken 2010). However, internal methods of self-financing, such as bootstrap financing, are of particular importance in times of great uncertainty. Crises are often characterized by a demand reduction as well as liquidity constraints, which in turn leads to an increased need for bootstrap financing.

While we were unable to identify prior research on the determinants of the use of bootstrap financing in crises, several studies indicate that the use of bootstrap financing measures is a critical tool for entrepreneurial ventures in terms of economic distress. For example, Winborg and Landström (2001) identify a cluster of ventures which they label “delaying bootstrappers.” These ventures are characterized by a business with a need for additional finances and use bootstrapping, for example, to delay different payments. They point out that such measures may serve as a relevant tool to survive as an entrepreneurial venture in crises. Ebben (2009) extends the study of Winborg and Landström (2001) and confirms that small firms that are illiquid or underperforming (“delaying bootstrappers”) use specific bootstrapping approaches more often. The author further argues that “empirical and anecdotal evidence suggests that small firms bootstrap out of necessity rather than proactively” (Ebben 2009, p. 349), which speaks to an increased usage of bootstrap financing in crises. Finally, Neeley and Van Auken (2010) explore gender differences in the use of bootstrapping within crises (i.e., the 2008/09 financial crisis). They argue that female business owners might be more likely to apply bootstrap financing since their business activities are probably more affected by a crisis than those of their male counterparts. This is in line with recent findings on the COVID-19 crisis, which also affects female entrepreneurs more severely (e.g., HÉTFA 2020).

## Hypotheses: determinants of bootstrap financing in crises

We investigate the use of bootstrap financing by self-employed individuals in crises. Specifically, we develop hypotheses about the determinants of bootstrap financing from three perspectives, namely, (1) a necessity, (2) human capital, and (3) an opportunity cost perspective.

### Necessity perspective

Even though the effects of the COVID-19 crisis on the business models and revenues of entrepreneurial ventures are generally negative, some heterogeneity exists. While some sectors flourish (e.g., online retailing), most ventures are negatively affected by the economic crisis. Therefore, we argue that entrepreneurial ventures that are more severely affected by the COVID-19 crises will more intensively look for opportunities since they face a necessity to ensure survival.

The preservation of liquidity, which is the main goal of bootstrap financing, is of crucial importance to ensure survival (e.g., Winborg and Landström 2001). Previous literature shows that entrepreneurs use bootstrap financing in crises to acquire necessary resources (Ebben 2009), although this often implicates the exploitation of others’ resources (Harrison et al. 2004; Malmström 2014). Since this hurdle of exploiting third parties exists, we argue that that entrepreneurial ventures who are more severely affected by the COVID-19 crisis are willing to engage in a greater amount of financial bootstrapping.

We assume that the degree of affectedness of a business within a crisis is determined by two factors. First, revenue decreases indicate the severity of the crisis for the respective entrepreneurial venture. If revenues decrease in a crisis and the costs remain at the same level, businesses face an immediate threat of bankruptcy and are thus forced to engage in bootstrap financing. Second, the amount of existing business liquidity determines how long the venture can overcome financial bottlenecks. Businesses need to maintain liquidity to meet short-term financial obligations. For example, employees need to receive their salary and monthly rents require payment. Thus, we assume that lower levels of liquidity in crises force ventures to more heavily engage in bootstrap financing. Our hypothesis is twofold:

- *A larger decrease in revenue is associated with an increased use of bootstrap financing measures in crises.*
- *A larger amount of business liquidity is associated with a decreased use of bootstrap financing measures in crises.*

### Human capital perspective

Our second set of hypotheses takes a human capital perspective and focuses on the entrepreneurial venture’s current CEO (i.e., the entrepreneur). Prior research in entrepreneurship and management shows that the CEO and their human capital characteristics are of crucial importance for firms’ strategic decisions (for a comprehensive review, see Liu et al. 2018). Similarly, prior entrepreneurship research shows that characteristics, such as entrepreneurial experience and education, matter for entrepreneurial behavior (e.g., Davidsson and Honig 2003; Robinson and Sexton 1994).

Prior bootstrapping research shows that entrepreneurial experience influences the likelihood of bootstrap financing (Winborg 2009). For example, Grichnik et al. (2014) show that entrepreneurs with higher managerial experience also use bootstrap financing more often. This is because these experiences provide them with better skills and a higher awareness of available resources. In line with this research, we argue that self-employed individuals with extensive entrepreneurial experience are more skilled in financial matters and have a better knowledge of the different financial instruments that exist to maintain liquidity. Hence, they should also have a higher likelihood to use bootstrap financing in crises in which the preservation of liquidity is of utmost importance. We hypothesize:

- *More self-employment experience of the entrepreneur is associated with an increased use of bootstrap financing measures in crises.*

Besides, various studies in entrepreneurial finance show that education and access to external financing are closely linked (e.g., Bates 1990; Carter et al. 2003; Coleman and Cohn 2000; Franke et al. 2006; Gartner et al. 2012). For example, Carter et al. (2003) demonstrate a positive relationship between the degree of education and financial resource acquisition of self-employed individuals. A frequent explanation is that individuals with a higher level of education, especially those with an academic education (i.e., a university degree), have greater cognitive capabilities and are more prone to change and adapting to uncertain environments. Furthermore, Grichnik et al. (2014) emphasize that academic education increases the likelihood to engage in bootstrapping. Since bootstrap financing has various facets, a higher degree of education should also be connected to a higher knowledge about different bootstrapping possibilities. Therefore, we assume that individuals with a higher level of education are also more likely to employ bootstrap financing activities in crises. Thus, we hypothesize the following:

H2b. *A higher level of education of the entrepreneur is associated with an increased use of bootstrap financing measures in crises.*

### Opportunity cost perspective

Our third set of hypotheses takes an opportunity cost perspective. Opportunity costs describe decisions that result in the loss of an available advantage through an alternative decision. Prior research explores different facets of entrepreneurship activities and opportunity costs (e.g., Amit et al. 1995; Kerins et al. 2004; Shane and Venkataraman 2000; Shane 2001) and utilizes opportunity costs to explain the loss of alternatives for self-employed individuals. For example, Amit et al. (1995) document a negative relationship between opportunity costs and entrepreneurial activities. Similarly, Lévesque and Minniti (2006) show that younger individuals more often start new businesses than older individuals due to different opportunity costs of time.

In line with this research, we argue that the entrepreneurial behavior of full-time and part-time entrepreneurs in crises should differ based on opportunity costs. Crises, such as the COVID-19 pandemic, increase the uncertainty associated with entrepreneurship. Accordingly, the opportunity cost of pursuing entrepreneurship increase for part-time entrepreneurs with a wage job next to their entrepreneurial venture. Of course, paid employment opportunities are also affected by the COVID-19 pandemic. This, however, should not be to the same extent as many governments like the German one help firms to maintain their jobs. Furthermore, part-time entrepreneurs often have the opportunity to switch to university, retire, or even take time off, which also increase the opportunity costs of pursuing entrepreneurship. As a result, individuals who are part-time self-employed are less willing to commit to and invest in an entrepreneurial career path. In contrast, full-time entrepreneurs mostly lack an available alternative within a crisis. Full-time entrepreneurs are more locked-in in their businesses since they have all their know-how bundled in the business. This leads us to the assumption that the use of bootstrap financing to maintain liquidity and manage venture survival becomes less likely for part-time entrepreneurs, while full time entrepreneurs will more heavily engage in bootstrap financing to ensure business survival. We hypothesize:

- *Compared to part-time self-employment, full-time self-employment is associated with an increased use of bootstrap financing measures in crises.*

Solo self-employed individuals account for a large and often underestimated proportion of all self-employed individuals (e.g., Lechmann and Schnabel 2014; Lechmann and Wunder 2017). They do not have employees and are also referred to as “own account workers” or “solo entrepreneurs” (e.g., De Vries et al. 2020; Van Stel and De Vries 2015). In Germany, for example, approximately 9% of the working population is self-employed (Eurostat 2019); solo self-employed individuals account for 50% of these individuals (Federal Ministry of Labour and Social Affairs 2018). However, there is a gap of knowledge of whether solo self-employed individuals behave differently in crises.

Regarding the opportunity costs in crisis, solo self-employed and their employer counterparts differ. Specifically, solo self-employed individuals have lower opportunity costs in those situations since those individuals have no employees to take care of and therefore lower hurdles to quit the business. Furthermore, self-employed individuals with further employees have a higher emotional involvement in their entrepreneurial ventures since they do not want their employees to lose their jobs (Yamakawa and Cardon 2017). Hence, they will be more willing to more heavily engage in bootstrap financing to preserve venture survival. We hypothesize:

H3b. *Compared to self-employment with employees, solo self-employment is associated with a decreased use of bootstrap financing measures in crises.*

Finally, prior research investigates how entrepreneurial behavior changes with increasing age (e.g., Azoulay et al. 2020; Kautonen et al. 2014; Kerr and Armstrong-Stassen 2011; Lévesque and Minniti 2006). A major factor for changing entrepreneurial behavior is the opportunity costs of time. Lévesque and Minniti (2006) thus demonstrate that aging goes along with a decreasing willingness to start a new venture due to higher opportunity costs of time. Also, handling information becomes harder with increasing age which can lead to reluctance to explore new avenues that require the gathering of new information (e.g., Bantel and Jackson 1989).

Thus, we argue that aging affects the use of bootstrap financing in crises. Since the value of time is higher and the risk propensity decreases (e.g., Hambrick and Mason 1984) if self-employed individuals get closer to retirement or if they are already retired, we assume that these individuals fight less hard for maintaining their business. Thus, we hypothesize:

H3c. *A higher age of the entrepreneur is associated with a decreased use of bootstrap financing measures in crises.*

## Context, data, and methods

### Context: the COVID-19 crisis in Germany

Our study is based on a primary dataset, which we collected via a survey in April and May, 2020. We conducted the survey while the COVID-19 crisis was in full effect in Germany and while Germanys’ economy was mostly locked down. Although Germany had a lower mortality rate than various other countries at this time (e.g., Bennhold 2020), over 130,000 people were already infected. The German government took many steps to contain the virus, which in turn had a severe impact on the German economy. At the time of the survey, a GDP decline between 4.5 and 9% is expected for the year 2020 (IFW Kiel 2020).

The German government introduced several supporting mechanisms to mitigate the economic consequences of the crisis. These measures included a crisis-related law regarding short-time work compensation, which allowed companies to maintain their workforce since Germany’s Federal Employment Office would pay workers at least 60% of their basic income (e.g., Taylor and Schwartz 2020). Also, Germany launched a €750 bn. economic aid package of which €50 bn. was directed to solo entrepreneurs and microenterprises (e.g., Nienaber 2020). Based on this program, self-employed individuals who were able to demonstrate acute liquidity shortfalls and that had up to five employees were able to receive immediate financial assistance of €9000, and those with up to ten employees were able to receive €15,000 for the following 3 months (Federal Ministry for Economic Affairs and Energy 2020). These governmental programs were necessary because initial evidence shows that many self-employed individuals faced severe declines in income (e.g., Bertschek and Erdsiek 2020; Kritikos et al. 2020).

**Fig. 1:**
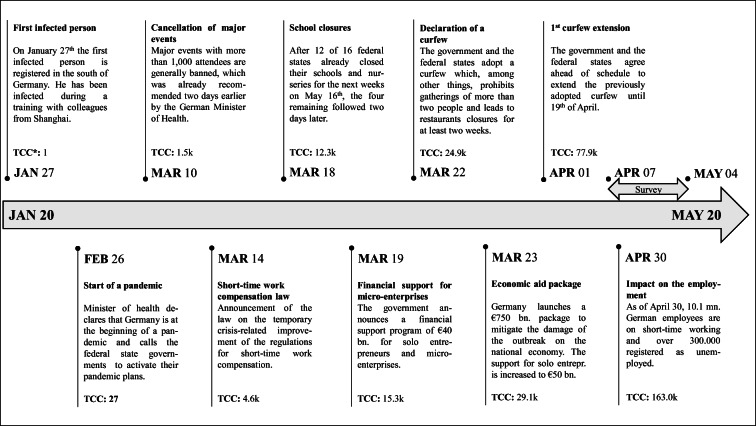
Major events during the beginning of the COVID-19 pandemic in Germany and survey period. *Notes*: * TCC = total confirmed cases by the John Hopkins University

Figure 1 describes the chronological sequence of the crisis and its consequences for Germany until our data collection was concluded. On January 27, the first person was contracted the coronavirus (e.g., Deutsche Welle 2020). Over the following months, different major events followed. For example, restaurants, shops, and schools were closed, and a curfew was imposed. On March 22, when the curfew was declared, 24,900 people had already been infected in Germany (John Hopkins University 2020). The curfew was partially loosened at the beginning of May.

### Sample and survey

Our online survey covered a broad range of topics related to COVID-19 and captured individuals’ characteristics, businesses’ characteristics, and the individual consequences of the pandemic. The survey was distributed via the Association of Founders and Self-employed Individuals in Germany (“Verband der Gründer und Selbstständigen Deutschland”, VDSG), which is one of the largest associations representing self-employed individuals in Germany. The VDSG sent personalized e-mails to its members and promoted the survey in its newsletters and through other associations representing self-employed individuals in Germany. Responses were collected between April 7 and May 4, 2020.

The survey was completed by 21,774 respondents. Due to the time-sensitive nature of the topic, we distributed the survey via a snowballing approach to maximize the number of responses. Therefore, we are unable to determine an accurate response rate. We excluded respondents with missing information for any of the variables considered in our analysis, which mainly resulted in dropout due to variables derived from questions that measured respondents’ wealth and business solvency. Such questions were not mandatory responses and were skipped by some respondents. The final estimation sample comprises 17,046 respondents (Table 1).

**Table 1:** Summarizes our variables and their definitions

Variable	Definition
*Dependent variable*	
Financial bootstrapping	Count variable that measures the number of bootstrapping measures used by the respondent. Responses include: (a) sending payment reminders, (b) paying invoices later, (c) reducing tax advances, (d) reducing health insurance contribution, (e) reducing commercial rent, (f) reducing private rent
*Independent variables*	
Revenue dummies	Set of dummy variables that capture the revenue change of the respondent’s business. Dummies include (a) stable/increased revenue or revenue decrease by (b) 100%, (c) 76 to 99%, (d) 51 to 75%, (e) 26 to 50%, and (f) 1 to 25% (serves as the reference category in the regression analysis)
Solvency dummies	Set of dummy variables that capture the solvency of the respondent’s business. Dummies include (a) insolvent, (b) solvent for 1 to 6 months, (c) solvent for 7 to 12 months, and (d) more than 12 months (serves as the reference category in the regression analysis)
Self-employment experience	Ordinal variable that captures the respondents self-employment experience in years (1 = 1–4, 2 = 5–10, 3 = 11–20, 4 = 21–30, 5 > 30)
Academic education	Dummy variable equal to one if the respondent received an academic education at university, zero otherwise
Full-time SE	Dummy variable that captures whether the respondent is working full-time (= 1) or part-time (= 0)
Solo SE	Dummy variable that captures whether the respondent is working solo (= 1) or with employees (= 0)
Age	Set of dummy variables that captures the age of the respondent. Dummies include (a) < 30 years, (b) 30–39 years, (c) 40–49 years, (d) 50–59, (e) > 59 years
*Control variables*	
Female	Dummy variable that captures whether the self-employed is female (= 1) or not (= 0)
Risk attitude	Ordinal variable that captures the individual’s risk attitude in entrepreneurial decision-making (1 = very low, 5 = very high)
Household size	Number of persons living in the respondent’s household (including the respondent), scaled from 1 to > 5
High digitalization	Dummy variable equal to one if the respondent describes the level of digitalization of his business as high or very high, zero otherwise
Mostly works from home	Dummy variable equal to one if the respondent mostly works from home, zero otherwise
Wealth	Ordinal variable that captures the respondent’s private wealth, that can be liquidated in the short term, in € (1 = 0; 2 = 30,000; 3 = 60,000; 4 = 90,000; 5 = 120,000; 6 = 150,000; 7 = 180,000; 8 = 210,000; 9 = 500,000; 10 = > 500,000)
Costs of living	Ordinal variable that captures the respondent’s private cost of living, in € per month (1 = 0–500, 2 = 501–1000, 3 = 1001-1500, 4 = 1501–2000, 5 = 2001–2500, 6 = 2501–3000, 7 = 3001–3500, 8 = 3501–4000, 9 = 4001–4500, 10 = 4501–5000, 11 = 5001–10,000, 12 = > 10,000)
Limited liability company	Dummy variable equal to one if the business is a limited liability company (i.e., UG, GmbH), zero otherwise
Government support	Dummy variable equal to one if the respondent received any governmental support during the Coronavirus crisis, zero otherwise
Region dummies	Set of 16 dummy variables that capture the location of the respondent’s business. The dummies refer to the 16 federal states of Germany
Industry dummies	Set of 15 dummy variables that capture the main industry of the respondent’s business. Dummies (a) IT, (b) engineering, (c) business services, (d) training, (e) proof-reading, (f) journalism, (g) culture, (h) event, (i) health and wellness, (j) tourism, (k) retail, (l) online retail, (m) finance and real-estate, (n) hospitality, and (o) others
Response week dummies	Set of five dummy variables that capture the week in which the respondent concluded the survey. Dummies refer to (a) calendar week 15 (06.04.–12.04.), (b) week 16 (13.04.–19.04.), (c) week 17 (20.04.–26.04.), (d) week 18 (27.04.–03.05.), (e) week 19 (04.05.–10.05.)

### Variables

#### Dependent variable

Our dependent variable captures the extent of bootstrapping measures employed by self-employed individuals. In the survey, we asked respondents whether they had implemented one or more of the following measures to preserve liquidity in response to the COVID-19 crisis: (a) sending payment reminders (warnings) to customers, (b) delaying payment on invoices that they received, (c) reducing tax advances, (d) reducing health insurance contributions, (e) reducing commercial rent, and (f) reducing private rent. The variable “bootstrapping measures” is a count variable and comprises the number of measures from the abovementioned list that were employed by the respondent, ranging from 0 to 6.

#### Independent variables

Our independent variables capture a range of individual, business, and COVID-19-related variables that might be related to the use of bootstrapping.

The first set of variables refers to H1a and measures the direct impact of the COVID-19 crisis on the respondents measured via the venture’s COVID-19 induced decrease in revenues. We construct a set of dummy variables indicating (a) a stable or even increased revenue as well as revenue decreases of (b) 100%, (c) 76 to 99%, (d) 51 to 75%, (e) 26 to 50%, and (f) 1 to 25% (reference category).

To measure H1b, we measure the venture’s solvency via a range of dummy variables. Specifically, we ask respondents to indicate how long their businesses would be able to maintain solvency given their current revenue and cost situation. The dummy variables are (a) already insolvent, (b) solvent for 1 to 6 months, (c) solvent for 7 to 12 months, and (d) solvent for more than 12 months (reference category).

To operationalize H2, we measure respondents’ self-employment experience by capturing the number of years that the individual has been self-employed in his or her current business (H2a). To account for the respondent’s level of education, we include a dummy variable equal to one if the respondent received an academic education at a university and zero otherwise (H2b).

To assess our opportunity cost-related hypotheses (H3), we capture whether the entrepreneur is working full-time or part-time (H3a). We also include a dummy variable that captures whether the entrepreneur is working solo or with employees (H3b). Finally, we measure the respondent’s age using an ordinal variable that is scaled from 1 (< 30 years) to 5 (> 59 years) (H3c).

#### Control variables

We include a range of control variables to account for confounding effects. To capture gender-related differences, we include a dummy variable that is equal to one for a female respondent and zero otherwise. To measure the association between bootstrapping and the propensity for risk-taking, we include a variable that captures the self-employed respondents’ attitudes toward risk. The variable ranges from 1 (very low) to 5 (very high). Additionally, we capture each respondent’s household size (including the respondent in this total). This variable is ordinal and scaled from 1 to “more than 5.”

Furthermore, we consider the businesses’ levels of digitalization (Bertschek and Erdsiek 2020). We ask respondents to indicate their business’ level of digitalization on a 5-point Likert scale. Based on this scale, we construct a dummy variable that is equal to one for respondents who stated that their businesses possessed either a high or a very high degree of digitalization. Since the bootstrapping measure of reducing corporate rent only applies to self-employed individuals who work in an outside office, we include a dummy variable that captures whether the respondent mostly works from home.

We include two variables that capture the respondents’ financial situations. First, we capture respondents’ private wealth, which might mitigate the need to implement bootstrapping measures. Specifically, we refer to the respondents’ assets that could be quickly liquidated to support the business. Possible responses ranged from 1 (€0) to 10 (more than €500,000). Second, we ask respondents to specify their monthly private cost of living. Possible responses range from 1 (€0–500) to 12 (more than €10,000).

To control for liability-related issues, we capture whether the respondent’s firm is incorporated as a limited liability company. For self-employed individuals in Germany, a limited liability company mainly refers to the corporation types “UG” and “GmbH.” Finally, we control for governmental support via a dummy variable that captures whether a self-employed has received governmental support or not.

The response to the COVID-19 crisis differed across the 16 German federal states. To capture these differences as well as the more general regional differences across Germany, we include a set of dummy variables that identifies the federal state in which the respondents’ business is located. To account for any industry-related differences, we include a set of 15 dummy variables that captures the main industry of the respondent’s business. Finally, the COVID-19 crisis developed rapidly, and the framework conditions for businesses frequently changed, for example, regarding government support and lockdowns. To capture differences related to the timing of respondents’ completion of the survey, we include a final set of five dummy variables that capture the week in which the respondent concluded the survey.

## Results

### Descriptive statistics and univariate analyses

Table 2 shows descriptive statistics and univariate analyses that provide initial insights into the relationship between our independent variables and the number of bootstrapping measures employed.

**Table 2:** Descriptive statistics and comparison of means

Column	(1)	(2)	(3)	(4)	(5)	(6)	(7)
Sample	Full sample				Subsamples: bootstrapping measures used		
					No	Yes	*t*/*z* test
N (observations)	17,056				4693	12,353	17,046
Variables	Mean	SD	Min	Max	Mean	Mean	Sig.
Dependent variable							
Financial bootstrapping	1.408	1.218	0	6	–	–	–
Independent variables							
Revenue stable/increase	0.002	0.042	0	1	0.004	0.001	
Revenue decrease: 100%	0.393	0.488	0	1	0.400	0.391	
Revenue decrease: 76–99%	0.245	0.430	0	1	0.200	0.262	***
Revenue decrease: 51–75%	0.168	0.374	0	1	0.161	0.170	
Revenue decrease: 26–50%	0.125	0.330	0	1	0.133	0.121	
Revenue decrease: 1–25% (ref.)	0.053	0.224	0	1	0.074	0.045	
Solvency: insolvent	0.112	0.315	0	1	0.105	0.114	
Solvency: 1–6 months	0.665	0.472	0	1	0.591	0.693	***
Solvency: 7–12 months	0.131	0.338	0	1	0.149	0.125	
Solvency: > 12 months (ref.)	0.092	0.290	0	1	0.154	0.069	***
Self-employment experience	2.662	1.142	1	5	2.572	2.695	***
Academic education	0.605	0.489	0	1	0.613	0.602	
Full-time SE	0.892	0.310	0	1	0.799	0.927	***
Solo SE	0.864	0.343	0	1	0.911	0.846	***
Age: 30–39	0.180	0.384	0	1	0.167	0.184	
Age: 40–49	0.277	0.447	0	1	0.248	0.287	*
Age: 50–59	0.369	0.483	0	1	0.369	0.369	
Age: > 60	0.138	0.345	0	1	0.167	0.127	*
Age: < 30 (ref.)	0.037	0.189	0	1	0.048	0.033	
Control variables							
Female	0.525	0.499	0	1	0.571	0.507	***
Risk attitude	3.048	0.967	1	5	2.966	3.079	***
Household size	2.195	1.124	1	5	2.149	2.213	***
High digitalization	0.169	0.375	0	1	0.162	0.172	
Mostly works from home	0.479	0.500	0	1	0.481	0.478	
Wealth	2.104	1.466	1	10	2.257	2.045	***
Costs of living	4.151	1.986	1	13	3.636	4.347	***
Limited liability company	0.041	0.198	0	1	0.030	0.045	
Government support	0.644	0.479	0	1	0.518	0.691	***

*Notes*: The significance levels displayed in column (7) refer to the results of a *t* test for ordinal/metric variables and to the results of a *z* test for proportions (i.e., dummy variables). * *p* < 0.05, ** *p* < 0.01, *** *p* < 0.001

Regarding our dependent variable “bootstrapping measures,” the results indicate that each self-employed individual used an average of 1.4 liquidity measures. However, this variable is skewed. Out of the 17,046 respondents in our final sample, 4693 (27.5%) did not use any bootstrapping measures. In contrast, 4987 (29.3%) respondents used one bootstrapping measure, 4351 (25.5%) used two, 2050 (12.0%) used three, 703 (4.1%) used four, 222 (1.3%) used five, and 40 (0.2%) respondents used all six measures. The most commonly used measure is reducing tax advances (47.5%, 8104 respondents) of all respondents, followed by reducing health insurance contributions (32.6%, 5555), sending payment reminders (25.7%, 4387), paying invoices later (16.9%, 2887), reducing private rent (9.9%, 1692), and reducing corporate rent (8.1%, 1376).

The descriptive statistics of our independent variables show that the category with the highest percentage of respondents (39.3%) suffered a total loss of revenues due to COVID-19. Additionally, 24.5% of the respondents reported a revenue decrease of 76–99%, and 16.8% of the respondents reported a decline of 51–75%. Collectively, 80.6% of the respondents reported revenue decreases of more than 50%, underlining the severe impact of the crisis on the self-employed individuals in our sample. Furthermore, the majority (66.5%) of the self-employed individuals indicate that they are only able to maintain solvency for the next 1 to 6 months. As mentioned above, we also asked respondents if they are full-time or part-time entrepreneurs and find that 89.2% are full-time self-employed. In addition, the dummy variable solo self-employed indicates that most respondents of our sample run their business alone (86.4%). Finally, our sample consists of 60.5% self-employed individuals with an academic background that are between 50 and 59 years old.

Table 2 also provides initial insights into how these characteristics vary with regard to the implementation of bootstrapping measures. Columns 5 and 6 show the mean values for each variable in the subsample of individuals who did not implement any bootstrapping measures (column 5) and the subsample of individuals who implemented at least one such measure (column 6). Column 7 displays the results of a *t*/*z* test for differences. Several significant differences emerge.

Regarding the individual characteristics, for example, the implementation of bootstrapping measures corresponds with lower age, more self-employment experience, a higher propensity to take risks, a larger household size, higher cost of living, and lower personal wealth. Further significant differences emerge regarding business solvency and the acceptance of government support.

Table 3 displays pairwise correlations and variance inflation factors. Overall, Table 3 suggests that multicollinearity does not seem to severely impact our results.

**Table 3:** Correlations and variance inflation factors (VIFs)

Variables	(1)	(2)	(3)	(4)	(5)	(6)	(7)	(8)	(9)	(10)	(11)	(12)	(13)	(14)	(15)	(16)	(17)	(18)	(19)	(20)	(21)	(22)	(23)	(24)	(25)	VIF
(1) Financial bootstrapping																										1.15
(2) Revenue stable/increase	− 0.03																									1.03
(3) Revenue decrease: 100%	0.01	− 0.03																								4.68
(4) Revenue decrease: 76–99%	0.07	− 0.02	− 0.46																							3.78
(5) Revenue decrease: 51–75%	0.01	− 0.02	− 0.36	− 0.26																						3.03
(6) Revenue decrease: 26–50%	− 0.05	− 0.02	− 0.30	− 0.21	− 0.17																					2.54
(7) Solvency: insolvent	0.07	− 0.01	0.14	− 0.03	− 0.06	− 0.06																				2.66
(8) Solvency: 1–6 months	0.09	0.00	−0.03	0.02	0.04	0.00	−0.50																			3.67
(9) Solvency: 7–12 months	− 0.07	0.01	− 0.04	0.00	0.00	0.04	− 0.14	− 0.55																		2.28
(10) Self-employment experience	0.04	− 0.03	− 0.02	0.02	0.00	0.00	− 0.06	0.02	0.02																	1.54
(11) Academic education	− 0.04	− 0.01	− 0.13	0.02	0.06	0.06	− 0.10	− 0.05	0.08	− 0.04																1.25
(12) Full-time SE	0.18	− 0.02	− 0.04	0.06	0.01	0.00	− 0.03	0.07	0.01	0.12	0.00															1.15
(13) Solo SE	− 0.13	0.00	0.05	0.00	− 0.02	− 0.04	0.03	− 0.06	0.01	− 0.06	0.13	− 0.10														1.41
(14) Age: 30–39	0.00	0.01	0.01	− 0.01	0.00	0.00	0.03	0.00	− 0.02	− 0.33	0.03	− 0.01	0.04													4.98
(15) Age: 40–49	0.05	− 0.01	− 0.01	0.00	0.01	0.01	0.00	0.01	0.00	− 0.09	0.01	0.04	− 0.02	− 0.29												6.81
(16) Age: 50–59	0.00	0.00	− 0.01	0.01	0.00	0.00	− 0.03	0.01	0.01	0.22	− 0.04	0.05	− 0.02	− 0.36	− 0.47											8.20
(17) Age: > 60	− 0.06	− 0.01	0.01	− 0.01	− 0.01	0.00	− 0.01	− 0.02	0.01	0.29	0.02	− 0.06	− 0.02	− 0.19	− 0.25	− 0.31										5.01
(18) Female	− 0.07	0.01	− 0.01	− 0.01	0.02	0.01	0.05	− 0.01	− 0.01	− 0.12	0.12	− 0.07	0.07	0.01	0.02	0.01	− 0.05									1.27
(19) Risk attitude	0.07	− 0.01	0.01	0.01	0.00	− 0.02	0.01	0.03	− 0.02	0.02	0.00	0.07	− 0.08	− 0.04	− 0.01	0.02	0.04	− 0.17								1.07
(20) Household size	0.02	0.01	− 0.05	0.01	0.02	0.03	− 0.03	0.01	0.00	− 0.02	0.00	− 0.01	− 0.12	− 0.02	0.17	− 0.02	− 0.13	− 0.08	0.00							1.13
(21) High digitalization	0.01	0.02	− 0.10	− 0.02	0.06	0.05	− 0.03	− 0.01	0.00	− 0.01	0.07	0.05	0.04	0.03	0.03	− 0.04	− 0.02	− 0.07	0.02	0.01						1.16
(22) Mostly works from home	− 0.03	0.02	− 0.14	0.03	0.06	0.06	− 0.05	− 0.03	0.03	− 0.05	0.19	0.01	0.21	0.03	0.00	− 0.01	− 0.02	0.03	0.01	− 0.04	0.20					1.22
(23) Wealth	− 0.10	0.01	− 0.06	0.02	0.02	0.01	− 0.22	− 0.24	0.18	0.07	0.14	0.05	− 0.03	− 0.05	− 0.01	0.04	0.05	− 0.09	0.00	0.05	0.06	0.08				1.48
(24) Costs of living	0.17	0.00	− 0.07	0.02	0.03	0.03	− 0.09	0.03	0.04	0.08	0.05	0.17	− 0.27	− 0.09	0.04	0.09	− 0.01	− 0.15	0.11	0.21	0.05	− 0.01	0.21			1.39
(25) Limited liability company	0.07	0.00	0.00	0.00	− 0.01	0.00	− 0.03	0.00	0.01	0.00	− 0.02	0.04	− 0.32	− 0.03	0.01	0.00	0.01	− 0.11	0.08	0.06	0.04	− 0.07	0.06	0.16		1.15
(26) Government support	0.19	− 0.04	0.08	0.09	0.00	− 0.09	0.06	0.13	− 0.07	0.06	− 0.10	0.22	− 0.17	− 0.01	0.01	0.01	− 0.02	− 0.06	0.09	− 0.01	− 0.06	− 0.09	− 0.14	0.06	0.05	1.25

*Notes*: Variance inflation factors (VIFs) are calculated based on the main regression model displayed in Table 4, column 4; *N* = 17,046

### Multivariate analyses

Our dependent variable “bootstrapping measures” is a count variable and ranges from 0 to 6. Because of the count nature of our dependent variable, we use a negative binomial regression model as our main form of analysis. The results are displayed in Table 4. We enter our sets of independent variables blockwise and jointly in column 4. The following interpretations refer to the full model (column 4).

**Table 4:** Main analysis: Results of a negative binomial regression model with the number of bootstrapping measures used as dependent variable

Column		(1)		(2)		(3)		(4)	
Statistic		Coeff.	(SE)	Coeff.	(SE)	Coeff.	(SE)	Coeff.	(SE)
Control variables									
Female		−0.055	(0.014)***	− 0.044	(0.015)**	− 0.053	(0.014)***	− 0.057	(0.015)***
Risk attitude		0.025	(0.007)***	0.030	(0.007)***	0.027	(0.007)***	0.022	(0.007)**
Household size		− 0.011	(0.006)	− 0.011	(0.006)	− 0.017	(0.006)**	− 0.015	(0.006)*
High digitalization		0.033	(0.018)	0.033	(0.018)	0.017	(0.018)	0.020	(0.018)
Mostly works from home		0.002	(0.014)	0.004	(0.014)	0.009	(0.014)	0.017	(0.014)
Wealth		− 0.037	(0.006)***	− 0.079	(0.005)***	− 0.081	(0.005)***	− 0.044	(0.006)***
Costs of living		0.065	(0.003)***	0.066	(0.003)***	0.055	(0.004)***	0.052	(0.004)***
Limited liability company		0.085	(0.030)**	0.069	(0.030)*	0.015	(0.031)	0.032	(0.031)
Government support		0.243	(0.015)***	0.302	(0.015)***	0.243	(0.015)***	0.182	(0.016)***
Independent variables									
Revenue stable/increase	*H1a*	− 0.424	(0.232)					− 0.369	(0.232)
Revenue decrease: 100%	*H1a*	0.222	(0.033)***					0.250	(0.033)***
Revenue decrease: 76–99%	*H1a*	0.315	(0.033)***					0.323	(0.033)***
Revenue decrease: 51–75%	*H1a*	0.271	(0.034)***					0.279	(0.034)***
Revenue decrease: 26–50%	*H1a*	0.179	(0.036)***					0.179	(0.036)***
Revenue decrease: 1–25% (ref.)	*H1a*	–	–						
Solvency: insolvent	*H1b*	0.509	(0.037)***					0.481	(0.037)***
Solvency: 1–6 months	*H1b*	0.380	(0.032)***					0.338	(0.032)***
Solvency: 7–12 months	*H1b*	0.218	(0.035)***					0.182	(0.035)***
Solvency: > 12 months (ref.)	*H1b*	–	–						
Self-employment experience	*H2a*			0.023	(0.006)***			0.039	(0.007)***
Academic education	*H2b*			0.004	(0.015)			0.024	(0.015)
Full-time SE	*H3a*					0.468	(0.028)***	0.437	(0.028)***
Solo SE	*H3b*					− 0.115	(0.020)***	− 0.123	(0.021)***
Age: 30–39	*H3c*					0.070	(0.039)	0.055	(0.040)
Age: 40–49	*H3c*					0.093	(0.039)*	0.054	(0.040)
Age: 50–59	*H3c*					0.024	(0.038)	− 0.034	(0.040)
Age: > 60	*H3c*					− 0.057	(0.041)	− 0.133	(0.044)**
Age: < 30 (ref.)	*H3c*					–	–	–	–
Region dummies (15)		Yes		Yes		Yes		Yes	
Industry dummies (14)		Yes		Yes		Yes		Yes	
Response week dummies (4)		Yes		Yes		Yes		Yes	
Observations		17,046		17,046		17,046		17,046	
Chi^2^		2081.619		1722.636		2127.848		2507.913	
Pseudo-R^2^		0.040		0.033		0.041		0.048	

*Notes*: * *p* < 0.05, ** *p* < 0.01, *** *p* < 0.001; *Coeff.* coefficient, *SE* standard error

We find support for our set of necessity hypotheses (H1), which indicates that the individuals’ severity of the crisis is associated with an increased amount of bootstrapping measures (H1a) while a higher amount of business liquidity is associated with a decreased use of bootstrap financing measures in crises (H1b). Thus, the larger the severity of the crisis for the entrepreneurial venture, the higher the amount of bootstrap financing activities.

Concerning our human capital hypotheses (H2), we find ambiguous results. On the one hand, we find support for a positive relationship between more self-employment experience and an increased use of bootstrap financing measures (H2a, *p* < 0.001). On the other hand, our results do not speak to H2b, which assumed that a higher level of education should be associated with an increased use of bootstrapping.

Finally, our results confirm our opportunity cost hypotheses. Specifically, full-time entrepreneurs apply a larger number of bootstrapping measures (H3a, *p* < 0.001). Also, solo self-employment is associated with a decreased number of bootstrap financing measures (H3b, *p* < 0.001). Finally, we find that especially the group of individuals with an age of over 60 years use a lesser amount of bootstrapping measures (*p* < 0.01), which speaks to the age effect hypothesized in H3c.

Regarding our control variables, the results show that women, a greater household size, and a larger personal wealth are associated with a decreased extent of financial bootstrapping measures used. Also, we find positive effects for risk attitude, costs of living, and the use of government support.

### Further analysis

As a further analysis, we disentangle the bootstrapping measures and analyze the use of each bootstrapping measure separately as a dependent variable (i.e., (1) sending payment reminders (warnings) to customers, (2) paying invoices later, (3) reducing tax advances, (4) reducing health insurance contributions, (5) reducing commercial rent, and (6) reducing private rent). Some of these measures are more COVID-specific than others. We would tentatively categorize (4) reducing health insurance contributions, (5) reducing commercial rent, and (6) reducing private rent as more COVID-specific. The results of the separate logistic regressions are shown in Table 5 and reveal some differences.

**Table 5:** Further analysis: Results of logistic regression models with specific bootstrapping measures as dependent variable

Column		(1)		(2)		(3)		(4)		(5)		(6)	
Dependent variable		Sending payment reminders (dummy)		Paying invoices later (dummy)		Reducing tax advances (dummy)		Reducing health insurance contr. (dummy)		Reducing commercial rent (dummy)		Reducing private rent (dummy)	
Statistics		Coeff.	(SE)	Coeff.	(SE)	Coeff.	(SE)	Coeff.	(SE)	Coeff.	(SE)	Coeff.	(SE)
Independent variables													
Revenue stable/increase	*H1a*	− 0.123	(0.431)	− 0.690	(0.751)	− 0.490	(0.483)	− 0.899	(0.744)	0.045	(1.040)	0.000	(.)
Revenue decrease: 100%	*H1a*	− 0.617	(0.079)***	0.035	(0.103)	0.748	(0.077)***	0.946	(0.088)***	0.525	(0.161)**	0.648	(0.167)***
Revenue decrease: 76–99%	*H1a*	0.076	(0.078)	0.296	(0.104)**	0.755	(0.078)***	0.800	(0.089)***	0.572	(0.163)***	0.452	(0.170)**
Revenue decrease: 51–75%	*H1a*	0.121	(0.080)	0.319	(0.106)**	0.552	(0.080)***	0.662	(0.092)***	0.419	(0.168)*	0.466	(0.174)**
Revenue decrease: 26–50%	*H1a*	0.210	(0.082)*	0.132	(0.112)	0.405	(0.083)***	0.307	(0.097)**	0.284	(0.175)	0.078	(0.187)
Revenue decrease: 1–25% (ref.)	*H1a*	–	–	–	–	–	–	–	–	–	–	–	–
Solvency: insolvent	*H1b*	0.109	(0.100)	1.649	(0.146)***	0.237	(0.088)**	0.579	(0.094)***	1.150	(0.186)***	1.671	(0.204)***
Solvency: 1–6 months	*H1b*	0.323	(0.076)***	1.025	(0.134)***	0.451	(0.070)***	0.373	(0.077)***	0.791	(0.169)***	0.826	(0.191)***
Solvency: 7–12 months	*H1b*	0.330	(0.082)***	0.365	(0.148)*	0.211	(0.076)**	0.203	(0.085)*	0.493	(0.182)**	0.139	(0.216)
Solvency: > 12 months (ref.)	*H1b*	–	–	–	–	–	–	–	–	–	–	–	–
Self-employment experience	*H2a*	0.075	(0.020)***	0.016	(0.023)	0.177	(0.018)***	0.020	(0.019)	− 0.027	(0.029)	0.008	(0.031)
Academic education	*H2b*	0.239	(0.043)***	0.013	(0.048)	0.038	(0.038)	0.040	(0.039)	− 0.135	(0.060)*	− 0.140	(0.064)*
Full-time SE	*H3a*	0.388	(0.070)***	0.006	(0.077)	0.758	(0.063)***	1.520	(0.084)***	− 0.104	(0.102)	0.038	(0.104)
Solo SE	*H3b*	− 0.169	(0.061)**	− 0.314	(0.066)***	− 0.346	(0.058)***	0.005	(0.059)	− 0.528	(0.075)***	0.214	(0.100)*
Age: 30–39	*H3c*	− 0.093	(0.102)	− 0.254	(0.119)*	0.362	(0.103)***	0.224	(0.104)*	0.475	(0.189)*	− 0.222	(0.143)
Age: 40–49	*H3c*	− 0.264	(0.103)*	− 0.088	(0.118)	0.457	(0.103)***	0.165	(0.104)	0.677	(0.187)***	− 0.368	(0.146)*
Age: 50–59	*H3c*	− 0.536	(0.106)***	− 0.197	(0.121)	0.314	(0.105)**	0.113	(0.106)	0.528	(0.190)**	− 0.480	(0.150)**
Age: > 60	*H3c*	− 0.811	(0.119)***	− 0.207	(0.133)	0.138	(0.114)	− 0.101	(0.117)	0.513	(0.202)*	− 0.615	(0.171)***
Age: < 30 (ref.)	*H3c*	–	–	–	–	–	–	–	–	–	–	–	–
Control variables		Yes		Yes		Yes		Yes		Yes		Yes	
Region dummies (15)		Yes		Yes		Yes		Yes		Yes		Yes	
Industry dummies (14)		Yes		Yes		Yes		Yes		Yes		Yes	
Response week dummies (4)		Yes		Yes		Yes		Yes		Yes		Yes	
Observations		17,046		17,046		17,046		17,046		17,046		17,016	
Chi^2^		1226.526		1266.687		2699.394		1395.863		1602.801		677.644	
Pseudo-R^2^		0.063		0.082		0.114		0.065		0.145		0.071	

*Notes*: * *p* < 0.05, ** *p* < 0.01, *** *p* < 0.001; *Coeff.* coefficient, *SE* standard error

The results show that the determinants of bootstrapping identified above differ in their effects regarding the specific bootstrapping measure used. While revenue decreases and low levels of business liquidity show effects with at least five of the six bootstrapping measures, other determinants such as self-employment experience are only correlated with some measures. The former result indicates that the most heavily affected self-employed individuals try almost everything to survive with their businesses. Interestingly, those self-employed individuals with high levels of experience make a clear distinction between customers, suppliers, and the state as stakeholders. While they are more likely than other self-employed individuals to send payment reminders to their customers (Column 1) and to reduce tax advances (Column 3), they are *not* more likely to delay their own payments. This could be interpreted as a form of solidarity among entrepreneurs or as a sign of maintaining a functioning entrepreneurial network and keeping good relations with suppliers. A similar pattern can be observed with full-time versus part-time self-employed individuals. Hence, it seems that entrepreneurial experience and entrepreneurial commitment do not only influence the *extent* of bootstrapping but also the *type* of bootstrapping.

## Discussion

### Interpretation of findings

Our results demonstrate that the severity of the crisis is closely connected to the extent of bootstrapping methods. This finding is in line with prior research that identifies liquidity issues as one of several bootstrapping drivers (e.g., Winborg and Landström 2001; Ebben 2009).

Furthermore, we find that self-employment experience has a positive effect on the use of bootstrapping measures. Thus, more experience as a self-employed provides an individual with greater knowledge of different bootstrapping methods, which in turn increases the options for them in crises. This finding is in line with Winborg (2009) but contrasts those of Grichnik et al. (2014) who find that managerial experience is an important bootstrapping driver, but entrepreneurial experience less so. The differences between their findings and our findings could be due to the different situations and samples involved. Our sample comprises many solo self-employed individuals, while their sample mainly comprises nascent entrepreneurs taking part in a business plan competition. Moreover, the entrepreneurs in our sample face a situation of urgent liquidity problems, while the entrepreneurs in their sample sought growth financing. This suggests that the determinants of bootstrapping are context dependent and differ from situation to situation. Another interesting finding relates to the type of bootstrapping measures used. Experienced self-employed individuals seem less likely than other self-employed to use bootstrapping measures that do harm to their entrepreneurial network. Additionally, we find that highly educated self-employed individuals do not differ from self-employed individuals with less education regarding the use of bootstrapping measures. Again, this finding contrasts the results of Grichnik et al. (2014) who find that a higher degree of education increases the use of bootstrap financing.

Finally, our results show that additional determinants can be explained from an opportunity cost perspective. First, we find that part-time self-employment, solo self-employment, and higher age are negatively associated with the number of bootstrapping measures. Due to an increased uncertainty in the COVID-19 crisis, opportunity costs increase more for part-time entrepreneurs. Therefore, they seem to fight less for the maintenance of their businesses. This finding is in line with previous literature that explores the relationship of opportunity costs and entrepreneurial activity (e.g., Amit et al. 1995; Kerins et al. 2004; Shane and Venkataraman 2000). Second, the negative effect of solo self-employment might be explained by a higher emotional involvement of these entrepreneurs. Third, we show that older self-employed individuals are less intensively engage in bootstrap financing than younger individuals. This negative effect is mainly due to the group of self-employed individuals older than 60 years. A tentative interpretation for this finding is that these self-employed individuals have a greater tendency to give up and are not as motivated as their younger counterparts due to higher opportunity costs of time. Consequently, they do not fight as hard as younger self-employed individuals to keep their businesses alive. This interpretation would be consistent with prior studies regarding the effects of age on entrepreneurial motivation (Lévesque and Minniti 2006). However, our results contrast these of Kautonen et al. (2014) who show that entrepreneurial activities increase in the sixties due to lower risks and a larger resource base of know-how.

Next to the opportunity cost theory, switching cost theory might partially explain these findings. Parker (2004) describes that higher switching costs increase the self-employed individuals’ likelihood to stay with the current occupation. In our case, part-time self-employed individuals might have lower switching costs due to lower lock-in effects. This finding is reinforced by Gohmann (2012), who shows that country-level institutions (e.g., level of economic freedom) affect switching costs and the prevalence of self-employment. Furthermore, solo self-employment also goes along with lower switching costs since they do not have responsibilities over employees. Finally, self-employed people who are over 60 years old may soon retire or are already retired, which potentially reduces switching costs makes them less dependent on bootstrap financing.

Our results also show that acceptance of government support and bootstrapping are complements and not substitutes. Controlling for the impact of the crisis and the individual liquidity situation of the self-employed individuals, we find that those who engage in bootstrapping measures also have a greater tendency to apply for government support. We interpret this finding as a sign of strong entrepreneurial motivation and argue that self-employed individuals who use both bootstrapping and apply for government support have a strong motivation to maintain their business through the crisis.

### Practical implications

Our study shows that self-employment experience has a positive effect on the use of bootstrapping. This finding suggests that government training, targeted entrepreneurship education programs, or webinars should focus on inexperienced entrepreneurs so that these individuals can be prepared to maintain liquidity in crises. The finding that government support and the use of bootstrapping measures are complementary also bears policy implications. It seems that government programs do not seem to crowd out the motivation of self-employed individuals to use their own measures to maintain their liquidity.

### Limitations and avenues for further research

The current COVID-19 crisis is an example of a situation of high uncertainty. When the respondents participated in our survey, it was unclear how long the crisis was going to last and how severe it would be. Hence, our results should be interpreted with great caution and cannot be generalized to all crisis situations that self-employed individuals may face. Another limitation concerns the nature of our sample since it comprises a majority of solo self-employed individuals, which is a large but specific group of entrepreneurs. They differ from other entrepreneurs in their financing behavior in that they rely mostly on internal financing instruments and that business, private life, and financing overlap to a large degree. Hence, our results should not be generalized to other groups of entrepreneurs, particularly those having employees and relying on external financing instruments such as bank loans or venture capital. Further research is needed in this regard.

Another important avenue for further research would be to investigate other determinants of bootstrapping in the COVID-19 crisis. For example, we would expect entrepreneurial motivation to play an important role. A significant share of solo self-employed individuals start their businesses out of necessity (De Vries et al. 2020) and are dependent self-employed (Román et al. 2011). Past research shows that necessity motivates entrepreneurial behavior and strategy (Block et al. 2015). Another determinant could be the social capital of solo self-employed individuals. Due to the general importance of social networks for entrepreneurship and entrepreneurial financing, we would expect these networks to have strong effects (Seghers et al. 2012). Finally, future research should also analyze performance effects and investigate whether and how bootstrapping in the COVID-19 crisis impacts long-term entrepreneurial performance and survival (Miao et al. 2017; Vanacker et al. 2011).
